# Correlation between Amerindian ancestry and neuromyelitis optica spectrum disorders (NMSOD) among patients in Midwestern Brazil

**DOI:** 10.1590/0004-282X-ANP-2020-0527

**Published:** 2022-06-24

**Authors:** Claudia Soares Alves, Flavia Borges Carapina Santos, Denise Sisterolli Diniz

**Affiliations:** 1 Universidade Federal de Goiás, Faculdade de Medicina, Centro de Referência em Doenças Desmielinizantes, Departamento de Neurologia, Goiânia GO, Brazil.

**Keywords:** Neuromyelitis Optica, Prevalence, Epidemiology, Neuromielite Óptica, Prevalência, Epidemiologia

## Abstract

**Background:**

Neuromyelitis optica spectrum disorder (NMOSD) is the second most frequently demyelinating, autoimmune, and inflammatory Central Nervous System (CNS) disease, and its prevalence varies greatly according to geography and ethnicity.

**Objective:**

To determine the prevalence and phenotype of NMOSD at a reference center for demyelinating diseases in Goiás State.

**Methods:**

This was a cross-sectional study, approved under CAAE number 8380.9317.9.0000.5078. All patients fulfilled the 2015 international consensus criteria.

**Results:**

Our study showed NMOSD as 9.37% of all demyelinating diseases registered in. It occurred predominantly in women (81%) and non-white individuals (83.4% had self-declared mixed skin color), and the median age at onset was 48 years. Amerindian ancestry was significantly higher (68.75%) than others. Longitudinally extensive transverse myelitis (LETM) alone ≥3 vertebral segments (35%) and optic neuritis (ON) alone (35%) were the most common onset manifestations. The median length of time from disease beginning to study enrollment was 48 months. A relapsing course and moderate disability (Expanded Disability Status Scale (EDSS) 3.0-4.0) were most commonly observed. The worst neurological impairments, characterized by EDSS>4.5, occurred more frequently in males (44.5% among men *versus* 20.5% among women). The majority of the patients had been receiving immunosuppressive treatment with azathioprine since the diagnosis of NMSOD: 77% (37) had a good therapeutic response. The prevalent outcome (84%) was permanent disability: 52% became physically handicapped; 54% had permanent visual impairment (25% with bilateral and 75% with unilateral amaurosis) and 30% had sphincter disability (82% with neurogenic bladder and 18% with ostomy).

**Conclusion:**

The estimated prevalence of NMOSD in Goiás is 0.79/per 100,000 inhabitants. The predominant phenotype comprises women, non-whites, onset in the fourth decade of life, relapsing course, and permanent moderate disability. Our study was the first on the epidemiology of NMOSD in Goiás, where NMOSD predominantly correlates with Amerindian ancestry.

## Resumo

**Antecedentes:**

O espectro da neuromielite óptica (ENMO) é a segunda doença desmielinizante, autoimune e inflamatória do sistema nervoso central, cuja prevalência varia conforme região geográfica e etnia.

**Objetivo:**

Determinar prevalência e fenótipo de ENMO em um Centro de Referência no estado de Goiás.

**Métodos:**

Estudo transversal, registrado no Certificado de Apresentação para Apreciação Ética (CAAE) sob o n° 8380.9317.9.0000.5078. Todos os pacientes preencheram os critérios do Consenso Internacional.

**Resultados:**

O ENMO representou 9,37% do total de doenças desmielinizantes. Foi predominante em mulheres (81%) e não brancos (83,4% autodeclararam-se mestiços), e a média de idade no momento de estudo foi 48 anos. A ancestralidade ameríndia foi significativamente maior (68,75%) que as demais. Mielite transversa longitudinalmente extensa isolada ≥3 segmentos vertebrais (35%) e neurite óptica isolada (35%) foram as apresentações iniciais mais comuns. A média de tempo entre o surgimento da doença e momento do estudo foi 48 meses. Predominaram: curso recidivante e incapacidade moderada (Expanded Disability Status Scale (EDSS) 3,0–4,0). Sequelas neurológicas caracterizadas por EDSS>4,5 predominaram em homens (44,5% entre estes *versus* 20,5% entre mulheres). Maioria em tratamento com azatioprina desde o diagnóstico, representando 77% (37 casos) de boa resposta terapêutica. Predominaram (84%) sequelas permanentes: 52% deficiência física permanente, 54% deficiência visual permanente (25% amaurose bilateral e 75% unilateral), e 30% deficiência esfincteriana permanente (82% bexiga neurogênica e 18% ostomias).

**Conclusão:**

A prevalência estimada de ENMO em Goiás é de 0,79/100.000 habitantes. Predominou o fenótipo mulheres, não brancos, no início na quarta década de vida, com curso recidivante, incapacidade permanente e moderada. Nosso estudo foi o primeiro em Goiás sobre a prevalência de ENMO e descobrimos que neste estado o ENMO predomina em pacientes com ancestralidade ameríndia (descendentes de nativos americanos).

## INTRODUCTION

Neuromyelitis optica spectrum disorder (NMOSD) is the second most frequent demyelinating disease of the Central Nervous System (CNS), after multiple sclerosis (MS)^1^. It has been known since 1894 when Devic published the first description of NMOSD, characterized by myelitis and optic neuritis^1,2^. Until 1999 NMOSD was misdiagnosed as MS, only this year when the first review of the clinical course of neuromyelitis optica disease was published it changed. In that review, it was recognized that NMOSD most frequently manifests with a relapsing course and that its clinical, laboratory, and imaging features allow it to be distinguished from MS^3^. A specific biomarker was discovered in 2004: a serum autoantibody immunoglobulin (IgG) against the aquaporin-4 (AQP4) water channel in the CNS, which was named AQP4-IgG or NMO-IgG^1,4^. The current international consensus on the diagnostic criteria for NMO was published in 2015 and unified the nomenclature to “neuromyelitis optica spectrum disorders” (NMOSDs) to encompass all the patients regardless of serological features^5^. The core clinical events refer to six neuroanatomically defined sites in the CNS: optic nerve, spinal cord, area postrema, brain stem, diencephalon, or cerebrum^1–5^. The most frequent clinical features of NMOSD are the following: strength failure (paraparesis or tetraparesis), sensitivity failure, loss of sphincter control, blindness, and intractable vomiting or hiccups^1–4^.

The prevalence of NMOSD has been reported to vary widely in different studies worldwide. It occurs most frequently among Asians and Afro-descendants^1–8^; women (70–90%) and non-whites, and the average age of onset is 40 years old^1,5^. The proportion of patients with NMOSD among all demyelinating disorders ranges from 1.2 to 39.3%, according to the geographical region^1–10^. NMOSD prevalence rates have been reported as 0.72-4.4/100,000 in Europe and 0.9 to 2.6/100,000 in Asia^1,2,6–8^. Across the Americas, extreme values have been described. The lowest was in Cuba: 0.52/100,000^9^; and the highest was in Martinique: 10/100.000^10^. In South America, the frequency of NMOSD is 11.8% among all demyelinating disorders, but the prevalence is unknown^11^. In a Brazilian series of patients, it was estimated that NMOSD represented 6-15% of all demyelinating diseases^12^. Because of the rarity of NMOSD and the lack of preclinical models, there are not enough cohort studies. Historically, NMOSD has been a difficult disease to study epidemiologically, and its prevalence has been estimated from reference center results^13^.

According to the national census of 2010, 46.7% of Brazilians identify themselves as being of mixed ancestry^14^. The state of Goiás, located in the Midwestern region, is in the “heart of Brazil” and surrounds the Brasília Federal District. It is the seventh-largest state in Brazil, with an area of approximately 340,126 km^2 14^. The prevalence of multiple sclerosis in the state of Goiás has been reported as 22.4/100,000^15^, but the prevalence of NMOSD in the state of Goiás remains unknown. According to an autosomal genetic study, the population of Goiás is derived from the miscegenation of three main ancestral groups: 3% native Amerindians, 83.7% Europeans (mainly Portuguese) and 13.3% Afro-descendants, typically from sub-Saharan Africa^16,17^.

This study aimed to evaluate the prevalence of NMOSD in the Goiás state, focusing on demographic characteristics in terms of ethnicity and ancestry, clinical manifestations, radiological and laboratory features, treatments, and outcomes.

## METHODS

This study was approved by the Research Ethics Committee of the Medical School of the Universidade Federal de Goiás, under CAAE number 8380.9317.9.0000.5078. Written and verbal consent was obtained from all subjects before the study procedures were started. This was a population-based descriptive cross-sectional study conducted at *Centro de Referência e Investigação em Esclerose Múltipla* (CRIEM), a demyelinating diseases reference center in Goiás state, located in Midwestern Brazil. Data were collected/patients were interviewed from 2017 to 2020.

Were included a total of 48 patients who fulfilled the International Panel for NMOSD Diagnosis 2015 criteria, and after they were classified according to their AQP4-IgG serostatus. Individuals fulfilling the inclusion criteria and without the exclusion criteria were eligible for enrollment. To accept the diagnosis of NMOSD, at least one specific core clinical criterion was needed among AQP4-IgG seropositive patients; or at least two specific core clinical criteria were needed when individuals were seronegative. Each patient's diagnosis required confirmation from and follow-up by at least two neurologists.

The main source of our information was a specific questionnaire which was filled out by one neurologist and reviewed by another neurologist, to determine participant eligibility. Our data were based on the hospital records and all the patients were interviewed and examined by the researcher group, to complete missing information. The first part of this questionnaire asked for general demographic information regarding the NMOSD patients, focusing on personal data: current age, gender, and ethnic categories, such as skin color and ancestry as far back as the third degree. The second part of this questionnaire was oriented toward personal medical history: age at disease onset, initial clinical event, number of relapses, and autoimmune comorbidities. We also requested information about other clinical comorbidities such as hypertension, diabetes, and thyroid dysfunction. Autoantibody seropositivity was defined as the detection of AQP4-IgG at any point during the participant's history and using any laboratory method. The presence of oligoclonal bands and antinuclear antibodies was also evaluated. All the radiological features, seen both on old and on current scans, were reevaluated by a group of neurologists, during the 24 months of this study.

We evaluated all the treatment strategies that were used to improve remission and prevent relapses, including associations of drugs at the acute phase, plasma exchange, and therapeutic failure to change the immunosuppressive drug. To describe the long-term outcome, we considered the total number of relapses, the classification according to Kurtzke, EDSS (Expanded Disability Status Scale), and the last follow-up. We subdivided all the disabilities into four groups: (1) permanent physical impairments (handicapped and needing bracing, wheelchair, or bedridden); (2) permanent visual impairment (unilateral or bilateral); (3) permanent sphincter deficiency (neurogenic bladder or ostomy); and (4) locked-in syndrome (permanent vegetative state).

Our numerical results are shown as means, percentages, and standard deviations. Nonparametric statistics with a direct counting method were performed and results were compared using the chi-square test or Fisher's exact probability test (when the criteria for the chi-square test were not fulfilled). *Odds Ratios* (ORs) were obtained to compare results. A p=0.05 was considered to be the limit of significance for allelic comparisons.

## RESULTS

According to the latest demographic Brazilian census, carried out in 2010, the population of the state of Goiás was 6,003,788 inhabitants, of whom 50.34% were women and 49.66% were men. We found 48 cases of people with NMOSD who were living in Goiás on the day of the prevalence study (October 1, 2020), which represented an estimated prevalence of 0.79/per 100,000 inhabitants. Also according to this demographic census, among the population of Goiás, 41% self-identified their skin color as white, 6.53% as black, 50.1% as mulatto (mixed race), and 0.14% as Amerindian ethnicity.

Regarding demyelinating diseases, 9.37% (48 cases) of the patients were diagnosed with NMOSD (Table 1). These individuals had a mean age of 43.29±15.51 years of age. There was a marked predominance of females (81.2%), and skin color was self-reported as white by 16.6%, black by 31.3%, and mulatto (mixed race) by 52.1%. Self-declared Amerindian ancestry was significantly higher (68.75 vs. 31.25% for other ancestries; p<0.039). The overall median age at NMOSD onset was 36.71±15.98 years of age, with a range from 9 to 66 years. The median length of time from disease onset to the time of study enrollment was 81.73±57.30 months.

**Table 1:** The phenotype of the patients with neuromyelitis optica spectrum disorders in Midwestern Brazil.

Demographic characteristics				Clinical manifestations		Radiological and laboratory features	
		Average±SD	Min-Max	Initial clinical event	Prevalence, %		Prevalence, %
Current age (years)		43.29±15.51	14.00–71.00	LETM+area postrema syndrome	2.1	Radiological cumulative features/ affected segments	
Age at onset (years)		36.71±15.98	9.00–66.00	LETM+brainstem syndrome	2.1	Orbit resonance/ optic neuritis	64.6
Time of disease (months)		81.73±57.30	8.00–240.00	LETM (alone)	35.4	Brain resonance	22.9
		**n**	**Prevalence, %**	**Simultaneous ON+LETM**	**20.8**	**Cervical spinal cord**	**43.8**
Gender				ON (alone)	35.4	Dorsal (thoracic) spinal cord	56.3
	Female	39	81.2	Area postrema syndrome	4.2	Lumbar spinal cord	16.7
	Male	9	18.8	Comorbidities		Laboratory features/ serological tests	
Skin color				Hypertension	27.1	AQP4-IgG serostatus positive	35.4
	White	8	16.6	Diabetes	10.4	Oligoclonal bands positive	22.9
	Black	15	31.3	Thyroid dysfunction	10.4	Antinuclear antibody (ANA) positive	22.9
	Mulatto	25	52.1	Most frequent autoimmune comorbidities		AQP4-IgG seropositivity adjusted prevalence	
Ancestry				Scleroderma	4.2	Female	88.2
	African	11	22.91	Lupus erythematosus	6.3	Male	11.8
	Amerindian	33	68.75	Sjögren disease	6.3	Amerindian ancestry	41.2
	European	4	8.34	Vitiligo	4.2	Other ancestries	58.8

NMOSD: neuromyelitis optica spectrum disorders; n: absolute requirement; %: relative frequency; SD: standard deviation; Mulatto: mixed skin color classification of people who are born with one white parent and one parent of another skin color; Amerindian: native indigenous ethnic groups of the Americas; LETM: longitudinally extensive transverse myelitis; area postrema: situated in the dorsal medulla oblongata, the lower part of the brainstem; it is a structure whose functions include control over vomiting and hiccups; ON: optic neuritis; AQP4-IgG: specific immunoglobulin for the water channel aquaporin-4.

**Figure 1:**
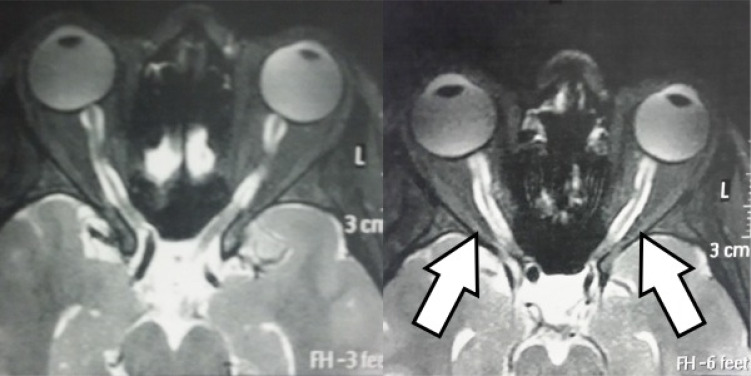
Orbital resonance showing bilateral longitudinally extensive optic neuritis. Axial section with T2-weighted acquisition.

Source: author's image bank.

**Figure 2:**
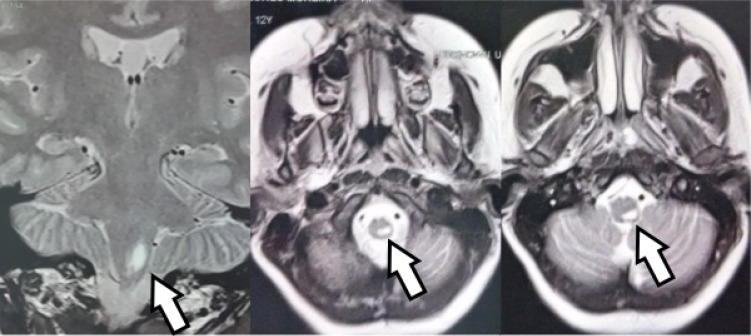
Brain resonance shows lesions in the brainstem and area postrema. Coronal and axial sections with T2-weighted acquisition.

Source: author's image bank.

**Figure 3:**
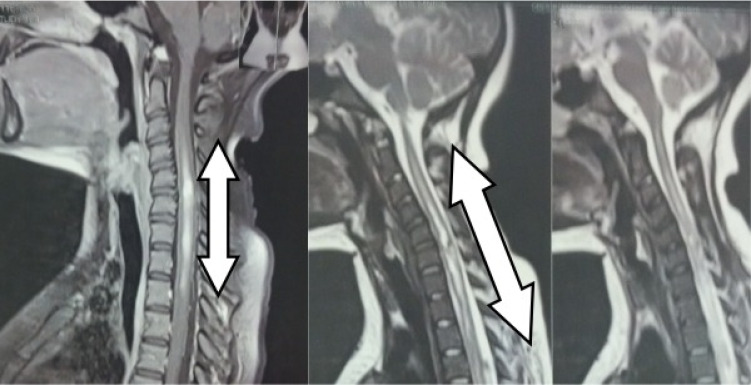
Spinal cord resonance showing longitudinally extensive transverse myelitis in cervical-thoracic segments (more than 3 vertebral segments). Sagittal section with T1 and T2-weighted acquisition.

Source: author's image bank.

The most common onset manifestations were longitudinally extensive transverse myelitis (LETM) alone on ≥3 vertebral segments (35.4%) or only optic neuritis (ON) (35.4%). The prevalences of the initial clinical event are also shown in Table 1 and Figures 1, 2, and 3. There was a predominance of a relapsing course: 72.9% of the individuals had 2-4 relapses (Table 2). The most frequent autoimmune comorbidities and systemic diseases were sclerodermas, lupus erythematosus, Sjögren disease, vitiligo, hypertension, diabetes, and thyroid dysfunctions (Table 1). No significant differences were found regarding serological status, gender, or ancestry (Table 1). The other serological tests were on antinuclear antibodies (22.9%) and the presence of oligoclonal bands in cerebrospinal fluid (22.9%).

**Table 2:** Treatments and outcomes among patients with neuromyelitis optica spectrum disorders in Midwestern Brazil.

Treatment strategies to improve remission and prevent relapses		Description and classification of the outcome and the permanent disabilities at last follow-up			
	%		%		%
Acute pharmacological treatment (onset or relapse)		Total number of relapses		Permanent physical deficiency	
Only high dose of IVMP	81.2	1 (monophasic course)	10.4	No physical impairments	60.4
Cyclophosphamide+IVMP	60.3	2 to 4	72.9	Mild handicapped - needs bracing	20.1
Intravenous immunoglobulin+IVMP	10.4	≥5	16.7	Moderate handicapped - wheelchair	35.4
Mycophenolate+IVMP	20.1	Long-term outcome		Severe handicapped - bedridden	20.1
Plasma exchange in acute treatment (in association with drugs)	20.8	Healthy	16.7	Permanent visual deficiency	
First maintenance treatment for preventing relapses in NMOSD		Impairments, limitations, and restrictions	77.1	No visual impairment	58.3
Beta-interferon or glatiramer (misdiagnosis as multiple sclerosis)	14.6	Death	60.2	Unilateral	33.3
Isolated prednisone	12.5	Classification according to EDSS at the last follow-up		Bilateral	80.4
Azathioprine	68.7	No impairment/ no disability (EDSS 0–1.5)	10.4	Permanent sphincter deficiency	
Rituximab	40.2	Mild disability (EDSS 2.0–2.5)	12.5	No sphincter impairment	77.1
Exchange of immunosuppressive therapy with a second drug		Moderate disability (EDSS 3.0–4.0)	45.9	Neurogenic bladder	18.7
Never changed the immunosuppressive drug (good response)	70.8	Severe disability/partial dependence (EDSS 4.5–6.5)	18.8	Ostomy	40.2
Therapeutic failure of the first drug and needed to change to azathioprine	14.6	Profound disability/total dependence (EDSS 7.0–9.5)	60.2	Locked-in syndrome	
Therapeutic failure of the first drug and needed to change to rituximab	14.6	Death caused by NMOSD (EDSS 10)	60.2	Permanent vegetative state	20.1

NMOSD: neuromyelitis optica spectrum disorders; IVMP: intravenous methylprednisolone; %: relative frequency; EDSS: Expanded Disability Status Scale (an outcome measurement that classifies demyelinating diseases, created by Kurtzke in 1983).

Magnetic Resonance Imaging (MRI) identified LETM spinal cord lesions in 35 cases (73%), and the cervicothoracic segment was the one most often affected. Spinal cord MRI showed that involvement of both the cervical and the dorsal spinal was significantly more common than involvement of only one of these two segments (40% both vs. 20% only cervical and 17% only dorsal). The cumulative frequency of segments affected in the spinal cord was 43.8% cervical, 56.3% dorsal, and 16.7% lumbar. Brain MRI examinations exhibited typical NMOSD in 22.9% of the patients. More than 64% of the NMOSD patients had optic nerve injuries (Table 1). The classical association between myelitis and optic neuritis, as first described by Devic in 1894, was found in 15 cases (31.3%).

In this study, disability in terms of EDSS was predominantly moderate (EDSS 3.0–4.0), which accounted for 45.9% of the cases; while 25% had worse classifications (EDSS 4.5–6.5 and 7.0–9.5) with limited mobility, as detailed in Table 2.

The majority of the patients had been on immunosuppressive treatment with azathioprine since receiving their diagnoses with NMOSD. Before the current NMOSD criteria, 14.6% of the patients had been misdiagnosed as presenting multiple sclerosis and received interferon and glatiramer, which worsened the clinical picture. After the NMOSD criteria definition, they were switched to azathioprine or rituximab, as detailed in Table 2.

Most of the patients (70.8%) never had any changes to their immunosuppressive drug, which means that a good therapeutic response had been achieved. We considered that a good therapeutic response consisted of the absence of new relapses, partial recovery, or improvement of EDSS. Despite the good therapeutic responses seen in this study, the diagnoses were made too late, and consequently, the specific immunosuppressive treatment was also started too late, after permanent impairments had already occurred (77.1%), and there was a death rate of 6.2%. The majority (84%) of the patients had developed permanent disability: 52% became physically handicapped, 54% acquired permanent visual impairment (25% with bilateral and 75% with unilateral amaurosis), and 30% developed sphincter deficiency (82% with neurogenic bladder and 18% with ostomy).

It was seen that 68.75% of the patients considered themselves to be Amerindians (descendants of Native Americans), while other ancestries accounted for the remaining 31.25%. Thus, the present study showed, for the first time, that there was a higher prevalence of this disease among descendants of the indigenous population (p<0.039) than among individuals of other ancestries.

## DISCUSSION

The classical worldwide epidemiological research described the overrepresentation of east Asians and Afro-descendants among patients with NMO/NMOSD^1,2,5,7,10,13^. The higher prevalence of Amerindian ancestry seen in our study conducted in the Brazil Midwestern region also differs from the findings of other Brazilian NMO studies which were conducted in other regions of this country of continental dimensions.

A study on genetic ancestry, conducted in 2013, which brought together data from five reference centers for demyelinating diseases in Brazil, showed that European ancestry was predominant in Brazil. However, that study^18^ also showed that the vast majority of patients with European ancestry were living in the southeastern region (80.5%; 87 cases), followed by the northeastern region (13%; 14 cases), while only 6.5% (7 cases) came from the state of Goiás, in the Midwestern region of Brazil. That study^18^, with a mixed group from different regions of Brazil, also identified that 68.7% were of European ancestry, 20.5% were of African ancestry and only 10.8% were of Amerindian ancestry. However, another study in the southeastern region of Brazil, showed that there was a predominance of Caucasians (54.9%) and that only 0.9% were Amerindians^14^. Principal component analysis has shown that groups of NMOSD patients in different Brazilian regions have differences in ancestry and phenotype^18^.

A literature review^19^ evaluated the phenotypic characteristics of the Brazilian NMO case series, covering the period from 2002 to 2014, five studies were found, out of which four were carried out in the southeastern region (two in Rio de Janeiro^20,21^ and two in São Paulo^22,23^) and one was carried out in the federal capital, Brasília^19^. All of these five studies described the predominance of NMOSD among individuals of non-white skin color, but none of them described their ancestry.

According to the national census of 2010, 46.7% of Brazilians identified themselves as being of mixed ancestry^14^. The state of Goiás, located in the Midwestern region, is at the “heart of Brazil” and surrounds the Brasilia federal district. It is the seventh-largest state in Brazil, with an approximate area of 340,126 km^2 14^. According to an autosomal genetic study, the population of Goiás is derived from miscegenation between three main ancestral groups: 3% native Amerindians, 83.7% Europeans (mainly Portuguese), and 13.3% afro-descendants, typically from sub-Saharan Africa^16,17^. The prevalence of multiple sclerosis in the state of Goiás had been reported to be 22.4/100.000^15^, but the prevalence of NMOSD remained unknown until now. Through the present research, it was shown that the estimated prevalence of NMOSD in Goiás was 0.79/per 100,000 inhabitants.

Although the predominance of HLA-DRB1*03 and DRB3 in mulattos with NMO/NMOSD in southeastern Brazil has been well described^24^, little is known about the possible HLA haplotypes of Amerindians, or about their correlations with the incidence and prevalence of this disease. Thus, the Amerindian genotype may resemble that of afro-descendants due to the high miscegenation of the Brazilian population, especially in the central-western region, where the proportions of Amerindians and blacks are high^14^.

Other epidemiological features shown among the NMO/NMOSD patients in the present study corroborate the results described by Flanagan et al.^10^, with a predominantly female (81.2%) and non-Caucasian (83.3%) population. The profile identified among patients who were followed up for 20 years of age at a reference center in São Paulo, southeastern Brazil, was that NMOSD accounted for 15.3% of demyelinating disorders^24^.

35% of the patients with NMOSD were positive for autoantibodies, with no significant differences in their concentrations between the groups. This proportion may have resulted from the following events: [I] 77% of the patients were taking azathioprine, which has the role of suppressing the immune response and may decrease the detection of autoantibodies through laboratory tests^1^; [II] the low sensitivity of the ELISA used in this study (60%) led to the occurrence of false negatives^25^; and [III] patients in the early stages of the disease may not yet have seroconverted or, because they are in the process of seroconverting, they may have low concentrations of AQP4-IgG, especially if the laboratory test used presented low sensitivity^26–30^. Thus, it is extremely necessary to improve the laboratory test for detection of AQP4-IgG that is used by the state government of Goiás.

In conclusion, this study reported on the demographics, clinical characteristics, treatments, and outcomes of neuromyelitis optica spectrum disorder (NMOSD) at a tertiary-level reference center in Goiás, Midwestern Brazil, over the last 24 months. A higher prevalence of this disease was found among Amerindian descendants. The estimated prevalence of NMOSD in Goiás was 0.79/per 100,000 inhabitants, and NMOSD accounted for 9.37% of the cases of demyelinating diseases seen in the reference center (CRIEM.) The predominant phenotype of NMOSD in Goiás consists of women, non-whites, the median age of onset in the fourth decade of life, relapsing course, and permanent moderate disability. These data may be useful in assessing the overall status of NMOSD among Amerindian descendants. Thus, studies on Amerindian HLA should be conducted to more accurately determine the reasons that lead to the higher prevalence of NMOSD among the descendants of native Americans.
